# Adaptation in the Alleyways: Candidate Genes Under Potential Selection in Urban Coyotes

**DOI:** 10.1093/gbe/evae279

**Published:** 2024-12-30

**Authors:** Samantha E S Kreling, Summer E Vance, Elizabeth J Carlen

**Affiliations:** School of Environmental and Forest Sciences, University of Washington, Seattle, WA 98195, USA; Department of Environmental Science, Policy, and Management, University of California–Berkeley, Berkeley, CA 94720, USA; Living Earth Collaborative, Washington University in St. Louis, St. Louis, MO 63130, USA

**Keywords:** adaptation, *Canis latrans*, evolution, urbanization

## Abstract

In the context of evolutionary time, cities are an extremely recent development. Although our understanding of how urbanization alters ecosystems is well developed, empirical work examining the consequences of urbanization on adaptive evolution remains limited. To facilitate future work, we offer candidate genes for one of the most prominent urban carnivores across North America. The coyote (*Canis latrans*) is a highly adaptable carnivore distributed throughout urban and nonurban regions in North America. As such, the coyote can serve as a blueprint for understanding the various pathways by which urbanization can influence the genomes of wildlife via comparisons along urban–rural gradients, as well as between metropolitan areas. Given the close evolutionary relationship between coyotes and domestic dogs, we leverage the well-annotated dog genome and highly conserved mammalian genes from model species to outline how urbanization may alter coyote genotypes and shape coyote phenotypes. We identify variables that may alter selection pressure for urban coyotes and offer suggestions of candidate genes to explore. Specifically, we focus on pathways related to diet, health, behavior, cognition, and reproduction. In a rapidly urbanizing world, understanding how species cope and adapt to anthropogenic change can facilitate the persistence of, and coexistence with, these species.

**Significance**

While the study of urban ecology has greatly expanded in the past two decades, we still have little work addressing the potential genomic effects of urbanization. For coyotes in particular, the ecological differences between urban and rural individuals have been well characterized, and numerous studies have begun addressing the neutral evolutionary questions of gene flow and genetic drift in urban areas. However, while we know there are genetic and ecological differences in urban coyotes, few studies have begun to look at specific genes or genome regions of interest that may be affected as a result of those genetic and ecological differences. Here, we present a detailed list of potential mechanisms and associated genes to facilitate future eco-evolutionary research on this adaptable urban canine.

## Introduction

Organisms living in cities experience novel disturbances that present challenges that are wholly unique relative to the environments they evolved in. In cities, organisms experience higher rates of interactions with people (Li and Wilkins 2014; Soulsbury and White 2014; Khan et al. 2018), exposure to novel food sources (Łopucki et al. 2021; Sarkar and Bhadra 2022), altered predator–prey interactions (Fischer et al. 2012; Eötvös et al. 2018), and environmental hazards including increased pollutant exposure and elevated temperatures (Lawson et al. 2011; Niemelä 2011; Da Silveira Felck et al. 2014). Although evolutionary ecologists have identified traits that are associated with success in establishing populations in cities (e.g. generalists vs. specialists; Rodewald and Gehrt 2014; Fisher and Burton 2018), our understanding of how the novel pressures of urbanization modify genomes is limited. This limitation is largely due to how difficult it is with current methodology to quantify evolution that increases fitness and disentangle it from neutral evolutionary processes (Lambert et al. 2021).

Cities are intrinsically heterogeneous landscapes with myriad selective pressures that have the potential to drive rapid evolution (Sih et al. 2011; Donihue and Lambert 2015; Alberti et al. 2017; Johnson and Munshi-South 2017; Santangelo et al. 2018; Diamond and Martin 2021; Lambert et al. 2021). Unfortunately, for many nonmodel species, evolutionary biologists cannot use typical methods (e.g. common garden experiments and knockout experiments) to prove adaptive evolution because many nonmodel taxa cannot be housed in common gardens, easily relocated, or housed in a laboratory. Additionally, making generalizable predictions about the directionality of selection in cities is challenging: some selection pressures may be heightened in urban areas (e.g. tolerance to pollutants; Whitehead et al. 2017), while others may be loosened (e.g. camouflage in response to predation; Kreling 2023). This is especially true since multiple selection pressure may have synergistic or antagonistic effects on evolution. For example, a species may deal with the urban heat island via thermoregulation adaptations and/or nocturnality. Moreover, heterogeneity within and between urban centers further complicates predictions. Due to these challenges, evolutionary biologists are just beginning to investigate potential genomic-level changes in relation to urbanization (Perrier et al. 2017; Mueller et al. 2018; Schell 2018; Salmón et al. 2021; Mascarenhas et al. 2022; Babik et al. 2023; Winchell et al. 2023; Caizergues et al. 2024).

Nevertheless, linking urban selective pressures with genomic changes is essential to our understanding of evolutionary ecology in a world of anthropogenic change. Whole-genome and epigenome sequencing are the gold standard for understanding evolutionary change and adaptation, but both methods can be cost-prohibitive, especially for nonmodel organisms. Although reduced-genome sequencing is less expensive, it still often exceeds wildlife-focused budgets, limiting the sample size and power of the study. For studies with limited budgets, targeting specific candidate genes for sequencing can allow testing of hypotheses while maintaining sufficient sample sizes to have statistical power.

Coyotes are widely distributed and incredibly successful in establishing urban populations throughout North America and have extensively studied natural history in both urban and nonurban systems alike (Hody and Kays 2018). Coyotes are also evolutionarily closely related to wolves and domestic dogs, with coyotes having split from wolves approximately 1 to 2 million years ago (Ersmark et al. 2016). Because the dog genome is incredibly well annotated, it can be levered in our study of coyotes (Lindblad-Toh et al 2005; Wilson and Rutledge 2021). Additionally, current and past hybridization with domestic dogs may have significant impacts on coyote evolution and behavior (Caragiulo et al. 2022). Thus, coyotes can serve as a blueprint for studying adaptive urban evolutionary processes in nonmodel, reference genome-less taxa. In this manuscript, we present candidate genes to investigate for adaptive evolution in urban coyotes via a variety of mechanisms related to diet, health, thermoregulation, behavior, cognition, and reproduction.

## Benefits and Limitations of a Candidate Gene Approach

The candidate gene approach involves researchers targeting particular genes of interest to sequence and compare. Candidate gene approaches are highly cost-effective allowing for large sample sizes and strong confidence in observed differences between groups. Both genetic and epigenetic candidate gene approaches also require basic lab work, making it within reach of many labs who may not specialize in genome-level work (Zhu and Zhao 2007). Candidate genes also allow researchers to leverage well-studied genomes of closely related species or highly conserved genes across taxa to hypothesize which genes may be involved in different traits or functions for nonmodel species. As in reduced-genome sequencing, a candidate gene approach cannot address pleiotropic or polygenic interactions, linkage disequilibria, or genetic drift (Shablin et al. 2015). These limitations must be taken carefully into consideration when drawing conclusions. Of note, genetic drift can readily be assessed prior to genomic studies using a variety of cost-effective methods such as single nucleotide polymophism (SNP) genotyping (Miles et al. 2019; Sommer 2020). Additionally, it is important to remember that observed phenotypic variation can be due to nongenetic factors such as plasticity, although there may still be genetic architecture that underlies this plastic capacity (Wong et al. 2005). Below we provide examples of which life history traits may be under selection in urban coyotes as well as a noncomprehensive list of candidate genes that have potential to be implicated based on current literature.

### Diet

Urban environments provide wildlife with a diverse set of food items that nonurban wildlife do not have access to (Birnie-Gauvin et al. 2017; Larson et al. 2020; Sugden et al. 2021). Additionally, anthropogenic food often has lower nutritional value but increased caloric density (Isaksson and Andersson 2007; Birnie-Gauvin et al. 2017), with low protein levels and high fat and carbohydrate levels (Murray et al. 2015). High carbohydrate diets have been linked to negative physiological outcomes for urban carnivores, including diabetes (as a result of increased sugar consumption), decreased insulin production (see Insulin Production and Regulation section), and changes in metabolic rates (see Metabolic Rate and Function section) (Schulte-Hoestedde et al. 2018; Strandin et al. 2018). Changes in diet have also been linked to numerous epigenetic changes such as DNA methylation that alter the metabolism of and response to different compounds and are semi-heritable (Weyrich et al. 2018; Chen et al. 2024). To deal with the high amounts of starches in anthropogenic foods, urban coyotes may undergo similar genetic changes as domestic dogs, which have adapted to living on high starch diets (see Starch Digestion section) (Axelsson et al. 2013; Arendt et al. 2014, 2016).

Moreover, increased dietary specialization as a result of more intraspecific competition among coyotes and greater diversity of food sources may also lead to increased morphological or physiological distinctions related to diet (Knudsen et al. 2009; Vamosi et al. 2014; DeSantis et al. 2022). With a greater number of potential diet items, individuals may be incentivized to specialize in particular foods to limit intra and interspecific competition (Bolnick et al. 2003). While there are often higher densities of mesocarnivores in urban areas compared to nonurban areas, predation rates by urban carnivores are significantly decreased as a result of anthropogenic food subsidies (Fischer et al. 2012; Eötvös et al. 2018). The need to hunt may be greatly reduced for urban coyotes (Eötvös et al. 2018), thus releasing predation-related traits (e.g. jaw strength/bite force) from historical evolutionary constraints. With less need to hunt natural prey and access to anthropogenic foods, urban coyote diets may become increasingly similar to the diets of domesticated dogs (Murray et al. 2015). Moreover, changes in diets are known to lead to morphological changes, and we propose urban coyote skull morphology may begin to look more similar to domestic animals (see Skull Shape section) (Schmitt and Wallace 2012; Marchant et al. 2017).

#### Insulin Production and Regulation

Insulin plays a fundamental role in regulating blood glucose levels and ensuring proper energy utilization by metabolizing carbohydrates (Rahman et al. 2021). Insulin facilitates the uptake of glucose, enabling cells to generate energy for vital physiological functions such as movement, reproduction, and thermoregulation (Rahman et al. 2021). Dysregulation of insulin results in health complications such as insulin resistance and hyperglycemia (Hess 2010). Urban raccoons (*Procyon lotor*) that consume human food tend to intake higher levels of sugar, which can lead to hyperglycemia (Schulte-Hoestedde et al. 2018). Similarly, yellow baboons (*Papio cynocephalus*) that consume higher amounts of garbage were found to have higher frequencies of insulin resistance (Banks et al. 2003). Domestic dogs commonly develop insulin resistance and eventually diabetes as a result of their high carbohydrate diets (Arendt et al. 2014). For urban coyotes, easy access to outdoor pet food and human refuse likely means higher consumption of glucose. If these sugar intakes are sufficient to cause insulin resistance and subsequent negative health outcomes, genes that help regulate insulin sensitivity and production may be selected for (Table 1).

**Table 1:** Candidate genes related to insulin production and regulation

Candidate gene	Gene location (domestic dog)	Gene function	Selection direction	Mechanism	References
G6PC2 (glucose-6-phosphatase catalytic subunit 2)	Primary_assembly 36: 13,839,759 to 13,847,446 forward strand. ROS_Cfam_1.0:CM025135.1	Encodes for protein associated with production of glucose and can act as an autoantigen which may increase susceptibility to diabetes.	Increased capacity for blood sugar regulation.	G6PC2 is associated with increased susceptibility to type 2 diabetes.	Al-Daghri et al. (2017)
GCK (glucokinase)	Primary_assembly 16: 14,738,733 to 14,780,998 forward strand. ROS_Cfam_1.0:CM025115.1	Encodes for proteins important in glucose metabolism pathways.	Increased capacity for blood sugar regulation.	Genetic variants of GCK are risk factors for type II diabetes.	Short et al. (2014)
GCKR (glucokinase regulator)	Primary_assembly 17: 21,755,097 to 21,780,825 forward strand. ROS_Cfam_1.0:CM025116.1	Encodes for receptors important in glucose metabolism pathways.	Increased capacity for blood sugar regulation.	GCKR may be associated with diabetes risk in domestic dogs.	Reiter et al. (2016)
HNF1A, HNF4A (hepatocyte nuclear factor 1 and 4 alpha homeobox A)	Primary_assembly 26: 17,072,065 to 17,091,161 forward strand. ROS_Cfam_1.0:CM025125.1 Primary_assembly 24: 32,536,281 to 32,612,159 forward strand. ROS_Cfam_1.0:CM025123.1	Encodes for transcription factors highly expressed in the liver.	Increased capacity for blood sugar regulation.	Unregulated diabetes can develop into congenital hyperinsulinism, diabetes mellitus, and diabetic nephropathies, all known to be associated with HNF1A and HNF4A genes.	Miyachi et al. (2022)

Listed from left to right are the names of the candidate gene and associated gene abbreviation, the location of the gene in the domestic dog genome as written in the Ensembl Genome Broswer, a brief synopsis of relative gene function, our hypothesized selective direction, the reasoning for why the gene may be under selection, and relevant references.

#### Metabolic Rate and Function

The metabolic cycle begins with nutrient processing from diet items, which leads to the chemical reactions within an organism that maintain life processes (e.g. energy production, growth, cell repair, and waste elimination; Kleiber 1961). Resting metabolic rate is the amount of energy consumed at rest (Norin and Metcalfe 2019). Energy management models suggest that increased risk-taking behavior should increase resting metabolic rate, and the pace-of-life hypothesis suggests that urban individuals should have higher metabolic rates (Réale et al. 2010). However, Oliveira et al. (2020) found that urban white-toothed shrews (*Crocidura russula*) had lower resting metabolic rate than their nonurban counterparts possibly due to differences in urban and nonurban environmental conditions, including lower prey availability and higher ambient temperature. Thus, selection may act upon genetic pathways that may alter resting metabolic rate, but the directionality is difficult to predict given conflicting findings of previous studies.

In mammals, metabolic function is highly influenced by the relationship between diet and the thyroid gland, fatty acids, and insulin (Eales 1988; Iwen et al. 2013). Thyroid glands release thyroid hormones, which play a large role in metabolism and are known to be genetically determined in humans (Panicker 2011). Thyroid disorders including hyperthyroidism and hypothyroidism are common in dogs (Singh and Beigh 2013) but poorly documented in wild canids. Fatty acids also influence the metabolic cycle via energy production and storage, growth, thermoregulation, and reproduction and are the building blocks to the fat stored in mammalian bodies (de Carvalho and Caramujo 2018). Fatty acid accumulation and composition are heavily influenced by diet, and urban wildlife is likely to have increased fat stores due to access to abundant food that is often high in saturated fats (Beckmann and Lackey 2008; Murray et al. 2015; Marechal et al. 2016; Lyons et al. 2018). A typical Western diet, which coyotes would have access to in most North American cities, has a very high ratio of omega-6 to omega-3 fatty acids. A high ratio of omega-6 polyunsaturated fatty acids can lead to strong proinflammatory responses that urban wildlife is likely experiencing as a result of diet (Isaksson et al. 2017; DiNicolantonio and O’Keefe 2021). An inflammatory response is part of the innate immune system that can cause significant oxidative stress and a negative fitness outcome (for more on oxidative stress, see Heavy Metals section in Immunology, Detoxification, and Thermoregulation section; Colitti et al. 2019). However, to our knowledge, no studies have examined if the metabolism of fatty acids has been altered in mammals across urbanization or what fatty acid composition looks like among urban mammals. Many genes govern fatty acid metabolism and variations in these genes, or in regulation of these genes, that increase efficiency in fatty acid metabolism and reduce oxidative stress may be important for urban coyotes (Table 2).

**Table 2:** Candidate genes related to metabolic rate and function

Candidate gene	Gene location (domestic dog)	Gene function	Selection direction	Mechanism	References
ACLS5 (acyl-CoA synthetase long chain family member 5)	Primary_assembly 28: 23,971,931 to 24,069,062 forward strand. ROS_Cfam_1.0:CM025127.1	Plays a key role in lipid biosynthesis and fatty acid degradation.	Increased fatty acid degradation efficiency Decreased fatty acid uptake	This gene plays a key role in the synthesis of lipids and the breakdown of fatty acids. In domestic dogs that have a deletion of this gene, individuals experience severe lipid malabsorption.	Mashek et al. (2006); O’Brien et al. (2020)

Listed from left to right are the names of the candidate gene and associated gene abbreviation, the location of the gene in the domestic dog genome as written in the Ensembl Genome Broswer, a brief synopsis of relative gene function, our hypothesized selective direction, the reasoning for why the gene may be under selection, and relevant references.

#### Starch Digestion

Outside of obligate carnivores, most mammals produce amylase (Boehlke et al. 2015) to help digest starches into smaller molecules such as maltose (Jacobsen et al. 1972). Access to anthropogenic resources in cities means coyotes are often eating carbohydrate-rich foods (Murray et al. 2015; Peyrot des Gachons and Breslin 2019), similar to the diet of domestic dogs (Murray et al. 2015). If this is the case, increased digestibility of starches may be selected for, as seen in domestic dogs who have increased copy number of AMY2B (Axelsson et al. 2013; Arendt et al. 2014), one of the genes responsible for amylase production and increased starch digestion efficiency (Table 3).

**Table 3:** Candidate genes related to starch digestion

Candidate gene	Gene location (domestic dog)	Gene function	Selection direction	Mechanism	References
ADGRE1 (adhesion G protein-coupled receptor E1)	Primary_assembly 20: 54,058,548 to 54,120,242 reverse strand. ROS_Cfam_1.0:CM025119.1	Production of G protein-coupled hormone receptors.	Increased capacity for blood sugar regulation.	In African wild dogs (*Lycaon pictus*) insulin secretion and sensitivity is associated with ADGRE1 in the adhesion G protein-coupled receptor family.	Liu et al. (2018)
AMY2B (pancreatic amylase)	Primary_assembly 6: 47,236,428 to 47,258,556 reverse strand.	Production of amylase.	Increased copy numbers	Higher copy numbers of these genes are associated with more efficient and complete digestion of starches. Domestic dogs have been found to have a 7-fold increase in the number of copies of the AMY2B gene compared to wolves. This change in copy numbers is thought to represent a significant shift in diet due to domestication.	Chatterton et al. (1996); Axelsson et al. (2013); Arendt et al. (2014)
MGAM (maltase-glucoamylase)	Primary_assembly 16: 7,045,909 to 7,120,389 reverse strand. ROS_Cfam_1.0:CM025115.1	Production of maltase-glucoamylase enzymes. Helps in the digestion of starches.	Increased starch digestion efficiency.	While examining amylase is essential to understanding how selection in urban environments may influence coyote diets, amylase is only one step of starch digestion. After, maltase-glucoamylase catalyzes the hydrolysis of maltose to glucose. While higher copy numbers of this gene have not been documented in domestic dogs compared to wolves, a specific haplotype (124 kb spanning the MGAM gene) is present in many domestic dogs. Most of the dogs tested in this study were homozygous for this haplotype, whereas the haplotype did not appear to exist in wolf populations. Since the maltase-glucoamylase gene helps in the breakdown of starches, it is likely that the high presence of this haplotype among domestic dogs indicates a potential selective advantage.	Axelsson et al. (2013)
SGLT1 (sodium/glucose co-transporter 1)	Primary_assembly 26: 25,271,492 to 25,344,793 forward strand. ROS_Cfam_1.0:CM025125.1	Regulates absorption and uptake of glucose	Increased starch digestion efficiency.	Domestic dogs were found to have a specific haplotype in the sodium/glucose cotransporter 1 gene which regulates the absorption and uptake of glucose. This haplotype is commonly found in domestic dogs but is relatively rare in wolves, indicating a selective advantage conferred by this haplotype in urban coyotes.	Wright et al. (2011); Axelsson et al. (2013)

Listed from left to right are the names of the candidate gene and associated gene abbreviation, the location of the gene in the domestic dog genome as written in the Ensembl Genome Broswer, a brief synopsis of relative gene function, our hypothesized selective direction, the reasoning for why the gene may be under selection, and relevant references.

#### Skull Shape

The sagittal crest is well developed in most mammalian carnivore species and provides surface area for increased muscle attachments (DeSantis et al. 2020; Coli et al. 2023). This increased muscle attachment allows for stronger bite force and assists in hard-object feeding (e.g. cracking bones; van Valkenburgh 2007). As a plastic response to decreased activity levels, altered masticatory muscle use, and altered diets, captive carnivores also often develop smaller sagittal crests (Washburn 1947; Brewer et al. 1994; Siciliano-Martina et al. 2021; Cooper et al. 2023), but even these plastic responses may be underlaid by alterations to epigenetic regulation. In domestic dogs, the sagittal crest is greatly reduced or even nonexistent (Schmitt and Wallace 2012), likely due to their more sedentary lifestyles and altered diets. While wild-captive comparisons suggest plastic environmental control of sagittal crest size, there may be genetic underpinnings as well (Cooper et al. 2022). For example, urban foxes were found to have extended posterior sagittal crests, but a reduced zygomatic region (i.e. cheekbone area; Parsons et al. 2020). The extended sagittal crest would suggest increased bite force, while a reduced zygomatic region suggests less developed masseter muscles (Parsons et al. 2020). These changes appear to primarily be the result of genetic changes rather than plasticity, although this was not explicitly tested through genomic means (Parsons et al. 2020). With the decreased need for bone cracking along with increased omnivory and soft-food feeding in cities (Murray et al. 2015), urban coyotes may lose sagittal crest definition, although this response could be plastic, genetic, epigenetic, or any combination therein. Alternatively, with an increased density of coyotes in urban areas, there may be an increase in intraspecific aggression, which would potentially favor increased bite force and sagittal crest height (Morin and Kelly 2017; Table 4).

**Table 4:** Candidate genes related to skull shape

Candidate gene	Gene location (domestic dog)	Gene function	Selection direction	Mechanism	References
SMOC2 (SPARC-related modular calcium binding 2)	Primary_assembly 1: 56,009,366 to 56,168,233 forward strand. ROS_Cfam_1.0:CM025100.1	Production of SPARC family protein important in embryogenesis.	Decreased sagittal crest height.	While we do not yet understand the genetics of sagittal crest development, research shows that the length of the skull is highly influenced by the SMOC2 gene in dogs and other canids. While this does not directly influence sagittal crest, alterations to the length of the skull can affect bite force and grip strength, which are similar to the effects of a decreasing or increasing sagittal crest height. This gene is also associated with skull and brain size.	Herring (2007); Marchant et al. (2017); Brassard et al. (2021)

Listed from left to right are the names of the candidate gene and associated gene abbreviation, the location of the gene in the domestic dog genome as written in the Ensembl Genome Broswer, a brief synopsis of relative gene function, our hypothesized selective direction, the reasoning for why the gene may be under selection, and relevant references.

### Immunology, Detoxification, and Thermoregulation

As hubs for industry, urban areas often have high air, soil, and water pollution from factories, vehicles, lead paint, and pesticides (see Heavty Metals section and Rodenticide Resistance section). These pollutants are known to cause or are correlated with numerous health afflictions (e.g. asthma, preterm births, and premature death) in nonhuman animal models and in humans (McDonnell et al. 1997; Tiryaki and Temur 2010; Da Silveira Felck et al. 2014; Zwolak et al. 2019; Johnson et al. 2024; Md Meftaul et al. 2020). Recent work has suggested urban pollutants may even have a cancerous effect on wild animals, where the prevalence of cancer is historically very low (Giraudeau et al. 2018; Pesavento et al. 2018; Sepp et al. 2019; Johnson et al. 2024). Frequent and prolonged contact throughout generations with carcinogenic materials may lead to selection for anticancer genes (Vittecoq et al. 2018; Boutry et al. 2020). Similarly, exposure to heavy metals and toxic chemicals can lead to lethal and sublethal negative fitness consequences including immune system depression (Namroodi et al. 2017; Serieys et al. 2018; Murray et al. 2019; Rodríguez-Estival and Mateo 2019). Thus, genes that allow for enhanced detoxification of the body or improved immune functioning may be valuable for urban wildlife (Reid et al. 2016; Whitehead et al. 2017; Vittecoq et al. 2018; see Innate Immunity section, Adaptive Immunity section, and Endocrine Disruptors section).

Additionally, urban areas are typically characterized by increased levels of impervious surfaces, such as concrete and asphalt, and a reduction of shade-providing trees (Arnfield 2003; Wang et al. 2019). This leads to regionally higher temperatures in urban areas, known as urban heat islands (Oke 1973; Arnfield 2003; Imhoff et al. 2010). People and wildlife in areas with high levels of impervious surface must cope with this increased temperature (see Thermoregulation section). We know that this heat increase is large enough to select for higher heat tolerance in ectotherms such as anoles (Campbell-Staton et al. 2020, 2021), but we have little idea how much urban heat islands affect endothermic wildlife species. Nevertheless, excessive heat takes a dramatic toll on human populations and over 10,000 people died of heat-related causes between 2004 and 2018 in the United States alone (Vaidyanathan et al. 2020). With such adverse effects on humans, it is likely that increased heat in urban areas has similarly adverse effects on other urban mammalian species and that genes associated with greater heat tolerance may be under selection.

#### Innate Immunity

In vertebrates, the innate immune system is the first line of defense against infection, reacting immediately in a nonspecific manner to foreign bodies (Riera Romo et al. 2016). Cities are characterized by higher pollution loads, increased contact with conspecifics, food provisioning (Strandin et al. 2018), and more interaction with domestic animals and their communicable diseases. Thus, urban coyotes are expected to have distinct innate immune systems when compared to their nonurban counterparts (DeCandia et al. 2019). While little work has been done outside of specific disease-state-associated innate immunity gene transcription, we expect genes that allow for advantageous innate immunity responses to be under positive selection in urban coyotes (Table 5).

**Table 5:** Candidate genes related to innate immunity

Candidate gene	Gene location (domestic dog)	Gene function	Selection direction	Mechanism	References
FOXA3 (forkhead box A3)	Primary_assembly 1: 110,281,781 to 110,290,035 reverse strand. ROS_Cfam_1.0:CM025100.1	Produces hepatocyte nuclear factors that act as transcription activators.	Increased efficiency in innate immune response.	Has been found to inhibit innate immune response to viral activity in asthmatic humans.	Chen et al. (2014); Stenz et al. (2019)
IVL (involucrin)	Primary_assembly 17: 62,860,777 to 62,861,634 forward strand. ROS_Cfam_1.0:CM025116.1	Produces cytoplasmic proteins.	Increased efficiency of innate immunity.	IVL is associated with skin barrier and pathogen recognition.	Burgess et al. (2010); Stoeckli et al. (2013); Kanwal et al. (2021)
NOD1/NOD2 (nucleotide binding oligomerization domain containing 1/2)	Primary_assembly 14: 43,105,475 to 43,194,265 reverse strand. ROS_Cfam_1.0:CM025113.1 Primary_assembly 2: 65,182,339 to 65,212,649 reverse strand. ROS_Cfam_1.0:CM025101.1	Stimulates immune reaction	Increased efficiency of innate immunity.	Recognizes molecules with bacteria peptidoglycan	Turchetti et al. (2015)
Nramp1 (natural resistance macrophage protein 1; synonym SLC11A1)	Primary_assembly 37: 25,053,097 to 25,063,084 forward strand. ROS_Cfam_1.0:CM025136.1	Stimulates immune reaction	Increased efficiency of innate immunity.	Reduces the pH of the phagosome making it toxic to bacteria.	Turchetti et al. (2015)
TLR1-7 (Toll-like receptors 1 to 7)	Primary_assembly 3: 74,315,975 to 74,325,027 forward strand. ROS_Cfam_1.0:CM025102.1	Encodes for a protein essential in pathogen recognition and activation on innate immunity.	Increased efficiency of innate immunity.	This class of genes is highly conserved from insects to vertebrates and has important effects on the innate immune system.	Huang et al. (2011)
TLR9 (Toll-like receptor 9)	Primary_assembly 20: 37,892,901 to 37,897,671 forward strand. ROS_Cfam_1.0:CM025119.1	Encodes for a protein essential in pathogen recognition and activation on innate immunity.	Increased efficiency of innate immunity.	This class of genes is highly conserved from insects to vertebrates and has important effects on the innate immune system.	Huang et al. (2011)
CBD103 (beta-defensin 103)	Primary_assembly 16: 57,070,481 to 57,083,181 forward strand. ROS_Cfam_1.0:CM025115.1	Partially controls pigment deposition. May have antimicrobial and immune system implications.	Increased efficiency of innate immunity.	CBD103 is associated with innate immunity.	Larsson and Tjälve (1978); Liu et al. (2004); Candille et al. (2007); Bridelli and Crippa (2008); Leonard et al. (2012); Chatelain et al. (2014); Kreling (2023)
PTPN6 (protein tyrosine phosphatase nonreceptor type 6)	Primary_assembly 27: 38,437,569 to 38,453,615 reverse strand. ROS_Cfam_1.0:CM025126.1	Encodes for a protein involved in cell signaling and regulator of hematopoietic cells.	Increased efficiency of innate and adaptive immunity.	PTPN6 is associated with increased inflammatory response and delayed healing. It is involved in both innate and adaptive immunity.	DeCandia et al. (2021); Kiratikanon et al. (2022)

Listed from left to right are the names of the candidate gene and associated gene abbreviation, the location of the gene in the domestic dog genome as written in the Ensembl Genome Broswer, a brief synopsis of relative gene function, our hypothesized selective direction, the reasoning for why the gene may be under selection, and relevant references.

#### Adaptive Immunity

The major histocompatibility complex is a crucial part of the mammalian adaptive immune system and is responsible for specific, targeted reactions to pathogens (Lukasch et al. 2017). In canids, the major histocompatibility complex is referred to as the dog leukocyte antigen (DLA) system. Across individuals, the major histocompatibility complex shows high genetic diversity, which allows populations to respond to a wide range of pathogens (Ujvari and Belov 2011). Flexible and diverse immune responses are especially important for urban populations that experience novel and/or increased toxin exposure and disease risk (Murray et al. 2019). For instance, bobcats in Los Angeles maintained high immunogenic variation while experiencing intense population decline, suggesting that diversity in a population's adaptive immunity may be essential for success in urban areas (Serieys et al. 2018). Alternatively, recently established coyotes in New York urban areas showed decreased major histocompatibility complex diversity compared to their nonurban counterparts, potentially a result of a founder effect (DeCandia et al. 2019). DeCandia et al. (2019) propose that the decrease in diversity may be a reflection of the short time that coyotes have lived in the New York City area, and thus this population has had less generational exposure to urban stress. Therefore, we expect long-established urban coyote populations to have increased immunogenic variation, while newly established populations may suffer from decreased variation. Of course, these trends are dependent on migration rates between urban and surrounding nonurban environments (Table 6).

**Table 6:** Candidate genes related to adaptive immunity

Candidate gene	Gene location (domestic dog)	Gene function	Selection direction	Mechanism	References
DLA-12, DLA-64, DLA-88 (dog leukocyte antigen)	Primary_assembly 12: 46,193 to 50,515 forward strand. ROS_Cfam_1.0:CM025111.1 Primary_assembly 12: 1,078,335 to 1,172,648 forward strand. ROS_Cfam_1.0:CM025111.1 Primary_assembly 12: 1,037,032 to 1,166,908 reverse strand. ROS_Cfam_1.0:CM025111.1	Part of the major histocompatibility complex. Involved in antigen recognition in the innate immune system.	Increased efficiency of innate immunity.	DLA genes are associated with adaptive immune responses and may be additionally upregulated during innate immune response in domestic dogs.	Miyamae et al. (2018)
PTPN6 (protein tyrosine phosphatase nonreceptor type 6)	Primary_assembly 27: 38,437,569 to 38,453,615 reverse strand. ROS_Cfam_1.0:CM025126.1	Encodes for a protein involved in cell signaling and regulator of hematopoietic cells.	Increased efficiency of innate and adaptive immunity.	PTPN6 is associated with increased inflammatory response and delayed healing. It is involved in both innate and adaptive immunity.	DeCandia et al. (2021); Kiratikanon et al. (2022)

Listed from left to right are the names of the candidate gene and associated gene abbreviation, the location of the gene in the domestic dog genome as written in the Ensembl Genome Broswer, a brief synopsis of relative gene function, our hypothesized selective direction, the reasoning for why the gene may be under selection, and relevant references.

#### Heavy Metals

Heavy metal toxicity generally occurs via the production of reactive oxygen species (Fu and Xi 2020). These reactive oxygen species damage cells and inhibit cellular metabolism, increase DNA methylation, and can result in cell death (Fridovich 2006; Ho et al. 2013; Phaniendra et al. 2015). Certain heavy metals can also replace essential metal ions, which serve as catalysts or activators for different enzymes (Jomova et al. 2024). This results in disruption of cell function, DNA damage, immune system suppression, and improper activation of certain transcription factors (Genestra 2007). Thus, genes that are involved in the production of enzymes or proteins that detoxify metals or reduce the impacts of oxidative stress on the body may be particularly beneficial for coyotes in urban areas facing increased exposure to heavy metals (Table 7).

**Table 7:** Candidate genes related to heavy metal detoxification

Candidate gene	Gene location (domestic dog)	Gene function	Selection direction	Mechanism	References
CBD103 (beta-defensin 103)	Primary_assembly 16: 57,070,481 to 57,083,181 forward strand. ROS_Cfam_1.0:CM025115.1	Partially controls pigment deposition. May have antimicrobial and immune system implications.	Increased melanin production for heavy metal detoxification and reduced oxidative stress.	Melanin molecules have negatively charged components, allowing them to bind to certain cations, including heavy metal cations. When incorporated into fur, these metal-bound melanins render those heavy metals inert. In urban environments where heavy metal exposure is high, producing more melanin may help detoxify the system and mediate negative fitness effects.	**Larsson and Tjälve (1978)**; Liu et al. (2004); Candille et al. (2007); Bridelli and Crippa (2008); Leonard et al. (2012); Chatelain et al. (2014); Kreling (2023)
MT-I through MT-IV (metallothionein-1 through 4)	MT-I: Primary_assembly 2: 60,142,510 to 60,149,806 reverse strand. ROS_Cfam_1.0:CM025101.1 MT-II: Primary_assembly 2: 60,152,673 to 60,154,757 reverse strand. ROS_Cfam_1.0:CM025101.1 MT-III: Primary_assembly 2: 60,169,544 to 60,171,119 reverse strand. MT-IV: Primary_assembly 2: 60,185,523 to 60,188,111 reverse strand. ROS_Cfam_1.0:CM025101.1	Production of metallothionein enzymes	Increased heavy metal detoxification and reduced oxidative stress.	The metallothionein I through IV genes are involved in the production of metallothioneins, enzymes that detoxify the body of heavy metals. MT-I and MT-II genes, in particular, are strongly conserved across mammalian species. Metallothioneins bind metals with thiol groups and help control oxidative stress by capturing free radicals. SNPs around the TATA box of the promoter in this gene cause variability in the quality of response to different heavy metals in humans. Importantly, these genes are often epigenetically activated by the presence of heavy metals or free radicals.	Hamer (1986); Andrews (2000); Ullio et al. (2015); Joneidi et al. (2019)
MTF1 (metal-regulatory transcription factor 1)	Primary_assembly 15: 4,808,870 to 4,846,324 forward strand. ROS_Cfam_1.0:CM025114.1	Regulates transcription of MT genes	Increased heavy metal detoxification and reduced oxidative stress.	While seemingly unstudied in canine cells, genetic changes in the regulators of MT genes in humans, such as metal-regulatory transcription factor 1, also appear to change quantity and quality of response to heavy metals.	Sims et al. (2012); Joneidi et al. (2019)
MC2R (melanocortin 2 receptor)	Primary_assembly 1: 24,287,716 to 24,288,606 forward strand. ROS_Cfam_1.0:CM025100.1	Production of eumelanin.	Increased eumelanin production for heavy metal detoxification and reduced oxidative stress.	See CBD103 mechanism.	Larsson and Tjälve (1978); Liu et al. (2004); Bridelli and Crippa (2008); Chatelain et al. (2014); Kreling (2023)
MLPH (melanophilin)	Primary_assembly 25: 48,506,902 to 48,555,712 forward strand. ROS_Cfam_1.0:CM025124.1	Produces melanophilin, a protein found in pigment cells.	Increased melanin production for heavy metal detoxification and reduced oxidative stress.	See CBD103 mechanism.	Larsson and Tjälve (1978); Liu et al. (2004); Bridelli and Crippa (2008); Chatelain et al. (2014); Kreling (2023)
TYRP1 (tyrosinase-related protein 1)	Primary_assembly 11: 34,222,689 to 34,240,515 forward strand. ROS_Cfam_1.0:CM025110.1	Encodes for tyrosinase related protein 1 which is implicated in melanogenesis.	Increased melanin production for heavy metal detoxification and reduced oxidative stress.	See CBD103 mechanism.	Larsson and Tjälve (1978); Liu et al. (2004); Bridelli and Crippa (2008); Chatelain et al. (2014); Kreling (2023)
ASIP (agouti)	Primary_assembly 24: 24,041,485 to 24,084,612 forward strand. ROS_Cfam_1.0:CM025123.1	Production of pheomelanin.	Increased pheomelanin production for heavy metal detoxification and reduced oxidative stress.	See CBD103 mechanism.	Larsson and Tjälve (1978); Liu et al. (2004); Bridelli and Crippa (2008); Chatelain et al. (2014); Kreling (2023)
CFAP20 (cilia- and flagella-associated protein 20)	Primary_assembly 2: 58,984,403 to 58,998,253 forward strand. ROS_Cfam_1.0:CM025101.1	Production of core inner junction proteins.	Increased melanin production for heavy metal detoxification and reduced oxidative stress.	See CBD103 mechanism.	Larsson and Tjälve (1978); Liu et al. (2004); Bridelli and Crippa (2008); Chatelain et al. (2014); Crystal et al. (2022); Kreling (2023)
IVL (involucrin)	Primary_assembly 17: 62,860,777 to 62,861,634 forward strand. ROS_Cfam_1.0:CM025116.1	Produces cytoplasmic proteins.	Increased efficiency of innate immunity.	IVL is associated with skin barrier and pathogen recognition.	Burgess et al. (2010); Stoeckli et al. (2013); Kanwal et al. (2021)
NOD1/NOD2 (nucleotide binding oligomerization domain containing 1/2)	Primary_assembly 14: 43,105,475 to 43,194,265 reverse strand. ROS_Cfam_1.0:CM025113.1 Primary_assembly 2: 65,182,339 to 65,212,649 reverse strand. ROS_Cfam_1.0:CM025101.1	Stimulates immune reaction	Increased efficiency of innate immunity.	Recognizes molecules with bacteria peptidoglycan	Turchetti et al. (2015)
Nramp1 (natural resistance macrophage protein 1; synonym SLC11A1)	Primary_assembly 37: 25,053,097 to 25,063,084 forward strand. ROS_Cfam_1.0:CM025136.1	Stimulates immune reaction	Increased efficiency of innate immunity.	Reduces the pH of the phagosome making it toxic to bacteria.	Turchetti et al. (2015)
TLR1-7 (Toll-like receptors 1 to 7)	Primary_assembly 3: 74,315,975 to 74,325,027 forward strand. ROS_Cfam_1.0:CM025102.1	Encodes for a protein essential in pathogen recognition and activation on innate immunity.	Increased efficiency of innate immunity.	This class of genes is highly conserved from insects to vertebrates and has important effects on the innate immune system.	Huang et al. (2011)
TLR9 (Toll-like receptor 9)	Primary_assembly 20: 37,892,901 to 37,897,671 forward strand. ROS_Cfam_1.0:CM025119.1	Encodes for a protein essential in pathogen recognition and activation on innate immunity.	Increased efficiency of innate immunity.	This class of genes is highly conserved from insects to vertebrates and has important effects on the innate immune system.	Huang et al. (2011)

Listed from left to right are the names of the candidate gene and associated gene abbreviation, the location of the gene in the domestic dog genome as written in the Ensembl Genome Broswer, a brief synopsis of relative gene function, our hypothesized selective direction, the reasoning for why the gene may be under selection, and relevant references.

#### Rodenticide Resistance

Indirect and direct exposure to rodenticides can have significant effects on carnivore health. Anticoagulant rodenticides interfere with vitamin K epoxide reductase and inhibit the formation of blood clotting factors in the liver (Watt et al. 2005). While lethal doses of this class of rodenticides cause animals to hemorrhage, they also have important sublethal effects, ranging from internal bleeding to reduced immune function, increased susceptibility to parasites and pathogens, and reduced fecundity (Kwasnoski et al. 2019; Quinn 2019). In response to high mortality rates, persistent rodenticide use has been selected for rodenticide resistance through multiple independent pathways in many species of rodents (Pelz et al. 2005; Ishizuka et al. 2008; McGee et al. 2020). Coyotes in urban and nonurban areas alike are often exposed to high levels of these rodenticides both through direct exposure and via bioaccumulation (McKenzie et al. 2022). If these sublethal, and occasionally lethal, effects have a strong enough impact on fecundity and fitness in urban coyotes, it may lead to selection for resistance to these compounds via epigenetic or genetic means (Table 8).

**Table 8:** Candidate genes related to rodenticide resistance

Candidate gene	Gene location (domestic dog)	Gene function	Selection direction	Mechanism	References
VKORC1 (Vitamin K epoxide reductase enzyme)	Primary_assembly 6: 17,271,517 to 17,273,431 forward strand. ROS_Cfam_1.0:CM025105.1	Production of vitamin K epoxide reductase enzyme.	Increased resistance to anticoagulant rodenticides.	For many anticoagulant rodenticides, resistance seems to stem from mutations within the vitamin K epoxide reductase gene, which encodes for an endoplasmic transmembrane protein. In dogs, we know this same gene is sensitive to these anticoagulant rodenticides such as warfarin. There has even been speculative selection for anticoagulant resistance for other carnivore species such as marten and ermine through the VKORC-1 gene.	Li et al. (2004); Choppin et al. (2009); Stöck et al. (2019)

Listed from left to right are the names of the candidate gene and associated gene abbreviation, the location of the gene in the domestic dog genome as written in the Ensembl Genome Broswer, a brief synopsis of relative gene function, our hypothesized selective direction, the reasoning for why the gene may be under selection, and relevant references.

#### Endocrine Disruptors

Endocrine-disrupting chemicals (e.g. solvents, plastics, and pharmaceutical agents) come in many forms and can interfere with a wide variety of the endogenous hormones found within the mammalian body, potentially altering growth, metabolism, and reproduction among other processes (Burkhardt-Holm 2010; Frye et al. 2012). Endocrine disruptors can act on genes directly but appear to more frequently induce epigenetic changes to DNA transcription and developmental mechanisms (Crews and McLachlan 2006). Endocrine-disrupting chemicals have been correlated with many diseases such as cancers, diabetes, thyroid disorders, and reproductive disorders in humans and domestic animals (Pocar et al. 2023). Unlike many mammals, it appears that domestic dogs are able to better metabolize and even eliminate certain persistent organic pollutants, a subclass of endocrine-disrupting chemicals (Pocar et al. 2023). Thus, in urban coyotes, endocrine-disrupting chemicals may select for specific pathways that regulate DNA transcription. For specific effects of environmental estrogens and androgens, a class of endocrine disruptors, on reproduction, see the Environmental Estrogens and Androgens section in Reproduction and Sexual Selection section.

#### Thermoregulation

Thermoregulation is the maintenance of an internal temperature conducive to an individual's physiological requirements (Romanovsky 2018). Endothermic species like mammals are capable of tolerating a wide range of environmental temperatures, but generally fare better with low body temperatures than with high body temperatures (Hansen 2009). When body temperature rises above homeostasis as a result of environmental conditions (i.e. heat stress), a number of negative physiological effects can occur in mammals including increased susceptibility to dehydration, metabolic disruptions, and compromised reproductive function via increased reactive oxygen species production (Hansen 2009; Fuller et al. 2020). Therefore, genes that control full-body responses to heat stress may be under selection. Bergman's law suggests that animals that face increased heat loads should become smaller in stature over time (Bergmann 1847). However, in a study on 100 North American mammals, urban animals were found to have larger body sizes compared to their nonurban counterparts (Hantak et al. 2021). Notably, this finding may not be genetic, but rather due to year-round access to anthropogenic foods that increase fat storage and quicken juvenile growth in urban wildlife (Hantak et al. 2021). Finally, darker coats often cause an increase in heat absorption but do not necessarily correlate to increased heat stress. However, if certain coat colorations, lengths, patterns, or textures confer better thermoregulation, they may be advantageous in urban environments (Kreling 2023). Thus, genes that influence responses to heat stress, body size, and coat color could be under selection in urban coyotes, but determining the strength and direction of selection will be dependent on the specific urban area and the temperature differences between this urban space and nearby nonurban spaces (Table 9). For information related to thermoregulation and reproduction please, see the Heat Stress section in Reproduction and Sexual Selection section below.

**Table 9:** Candidate genes related to thermoregulation

Candidate gene	Gene location (domestic dog)	Gene function	Selection direction	Mechanism	References
CBD103 (beta-defensin 103)	Primary_assembly 16: 57,070,481 to 57,083,181 forward strand. ROS_Cfam_1.0:CM025115.1	Partially controls pigment deposition. May have antimicrobial and immune system implications.	Increased or decreased melanin production for thermoregulation.	If certain colorations confer better thermoregulation, genes controlling melanin placement and production may be affected.	**Larsson and Tjälve (1978)**; Liu et al. (2004); Candille et al. (2007); Bridelli and Crippa (2008); Leonard et al. (2012); Chatelain et al. (2014); Kreling (2023)
FGF5 (fibroblast growth factor 5)	Primary_assembly 32: 4,532,937 to 4,554,671 forward strand. ROS_Cfam_1.0:CM025131.1	Production of fibroblast growth factor 5, implicated in transition of hair follicles	Decreased fur length. Increased thermotolerance.	Associated with different coat textures and lengths. If a specific coat texture such as curly, short, or long fur is advantageous for thermoregulation, it may be selected for in urban regions.	Maeda et al. (2007); Zhang et al. (2020)
HSF1 (heat shock transcription factor 1)	Primary_assembly 13: 38,200,916 to 38,221,478 forward strand. ROS_Cfam_1.0:CM025112.1	Production of heat shock protein often in response to temperature stress.	Increased thermotolerance.	In response to high heat events, this transcription factor up or downregulates 1,500 to 8,000 different genes. Variations in HSF1 that confer more efficient or effective responses to higher heat loads may thus be selected for in urban populations, especially as extreme heat events become more frequent under changing climate regimes.	Stone et al. (2010)
IGF1 (insulin-like growth factor 1)	Primary_assembly 15: 41,856,552 to 41,930,595 reverse strand. ROS_Cfam_1.0:CM025114.1	Production of the hormone insulin-like growth factor 1. Involved in body size determination across mammalian species.	Decrease in body size.	IGF1 mediates the effects of growth hormone and stimulates bodily growth via the transduction of a tyrosine kinase signal. Serum levels of IGF1 are often correlated to body size in mammals.	Favier et al. (2001); Sutter et al. (2007); Zapata et al. (2016); Plassais et al. (2019)
IRS4 (insulin receptor substrate 4)	Primary_assembly X: 83,925,941 to 83,942,143 reverse strand. ROS_Cfam_1.0:CM025138.1	Encodes for cytoplasmic protein that acts as an insulin receptor.	Decrease in body size.	Bergmann's rule predicts that animals in warmer areas should have smaller body sizes. Thus, in cities, we may expect to see a reduction in body size to assist in thermoregulation.	Plassais et al. (2017)
KRT7 (keratin 7)	Primary_assembly 27: 2,656,611 to 2,826,996 reverse strand. ROS_Cfam_1.0:CM025126.1	Produces neutral proteins and is often expressed in epithelial tissues.	Decreased fur length. Increased thermotolerance.	See FGF5 mechanism.	Zhang et al. (2020)
MC2R (melanocortin 2 receptor)	Primary_assembly 1: 24,287,716 to 24,288,606 forward strand. ROS_Cfam_1.0:CM025100.1	Production of eumelanin.	Increased or decreased eumelanin production for thermoregulation.	See CBD103 mechanism.	Larsson and Tjälve (1978); Liu et al. (2004); Bridelli and Crippa (2008); Chatelain et al. (2014); Kreling (2023)
MLPH (melanophilin)	Primary_assembly 25: 48,506,902 to 48,555,712 forward strand. ROS_Cfam_1.0:CM025124.1	Produces melanophilin, a protein found in pigment cells.	Increased or decreased melanin production for thermoregulation.	See CBD103 mechanism.	Larsson and Tjälve (1978); Liu et al. (2004); Bridelli and Crippa (2008); Chatelain et al. (2014); Kreling (2023)
RSPO2 (R-spondin 2)	Primary_assembly 13: 8,874,544 to 9,021,570 reverse strand. ROS_Cfam_1.0:CM025112.1	Production of R-spondin 2 ligand that is a transducer.	Decreased fur length. Increased thermotolerance.	See FGF5 mechanism.	Zhang et al. (2020)
TYRP1 (tyrosinase related protein 1)	Primary_assembly 11: 34,222,689 to 34,240,515 forward strand. ROS_Cfam_1.0:CM025110.1	Encodes for tyrosinase related protein 1, which is implicated in melanogenesis.	Increased or decreased melanin production for thermoregulation.	See CBD103 mechanism.	Larsson and Tjälve (1978); Liu et al. (2004); Bridelli and Crippa (2008); Chatelain et al. 2014; Kreling 2023
ASIP (agouti)	Primary_assembly 24: 24,041,485 to 24,084,612 forward strand. ROS_Cfam_1.0:CM025123.1	Production of pheomelanin.	Increased or decreased pheomelanin production for thermoregulation.	See CBD103 mechanism.	Larsson and Tjälve (1978); Liu et al. (2004); Bridelli and Crippa (2008); Chatelain et al. (2014); Kreling (2023)
CFAP20 (cilia- and flagella-associated protein 20)	Primary_assembly 2: 58,984,403 to 58,998,253 forward strand. ROS_Cfam_1.0:CM025101.1	Production of core inner junction proteins.	Increased or decreased melanin production for thermoregulation.	See CBD103 mechanism.	Larsson and Tjälve (1978); Liu et al. (2004); Bridelli and Crippa (2008); Chatelain et al. (2014); Crystal et al. (2022); Kreling (2023)

Listed from left to right are the names of the candidate gene and associated gene abbreviation, the location of the gene in the domestic dog genome as written in the Ensembl Genome Broswer, a brief synopsis of relative gene function, our hypothesized selective direction, the reasoning for why the gene may be under selection, and relevant references.

### Cognition and Neuroanatomy

Cognition refers to the mechanisms by which animals acquire, process, store, and act on information from the environment (Shettleworth 2010), enabling animals to assess risks, make decisions, disperse, obtain resources, and avoid mortality. The cognitive buffer hypothesis posits that large brains have evolved to facilitate cognitive abilities, like learning and problem solving (Aiello and Wheeler 1995; Sol 2009a, 2009b). As a result of increased novelty and environmental complexity, animals in urban environments may need to be more effective learners and problem solvers (Møller 2009) and may develop greater cranial capacity compared to their nonurban counterparts (Snell-Rood and Wick 2013; see Brain Size section). Urban colonization is even predicted by bird species with larger relative brain sizes but has not been studied in mammals (Carrete and Tella 2011; Maklakov et al. 2011). Alternatively, cognitive needs may be reduced in urban areas as a result of decreased predation rates and increased food supplementation (Lesch et al. 2022; Vincze and Kovács 2022). As such, changes in cognition are often species and context specific. Nevertheless, changes associated with urban living, whether it be to enhance or reduce cognitive complexity (see Problem Solving, Memory, and Learning section), may exert selection on genes that influence neural development, brain size, and skull morphology (Parsons et al. 2020), plasticity (Pearson-Fuhrhop and Cramer 2010; von Bernhardi et al. 2017; see Cognitive Plasticity section), and other abilities like learning, memory, and problem solving (Carrete and Tella 2011; Maklakov et al. 2011; Snell-Rood and Wick 2013). However, research on the cognition and neuroanatomy in wildlife is lacking and there is still much to learn about the brain, cognitive abilities, and what role genetics play in shaping how brains function (Griffin et al. 2017; Goumas et al. 2020). It should also be noted that the brain is a highly plastic organ and that gene expression and pleiotropy are critical for cognitive processing (Trzaskowski et al. 2013; Mathias et al. 2023). Thus, in urban coyotes, selection for cognition-related traits may not be as pronounced as other morphological traits, and which genes are under selection in addition to the direction of selection may be difficult to predict (Vincze and Kovács 2022).

#### Brain Size

Brain size is correlated with establishment in novel environments; thus, brain size may be selected for in cities, which confront animals with novel challenges that they must adjust to for survival (Snell-Rood and Wick 2013). On the other hand, reduced predation pressure and easier access to food via anthropogenic sources may reduce the need for large cranial capacity (Snell-Rood and Wick 2013), making the directionality of selection difficult to predict. While little research has been done on urban mammalian cranial capacity, Parsons et al. (2020) found that urban red foxes (*Vulpes vulpes*) in London, England tended to have skull traits more similar to domesticated canids, including reduced sexual dimorphism, shorter and wider muzzles, and smaller braincases (Parsons et al. 2020). However, their sampling was not spatially discrete, and more work should be done to confirm if these findings were truly a result of urbanization. Meanwhile, Siciliano-Martina et al. (2022) found endocranial volume (a proxy for brain size) rose over successive captive generations and hypothesized that this increased volume was due to the high-quality nutrition provided while in captivity. Thus, for urban coyotes, we may expect to see braincases change in size, but the directionality is difficult to predict and likely varies by context (Table 10).

**Table 10:** Candidate genes related to brain size

Candidate gene	Gene location (domestic dog)	Gene function	Selection direction	Mechanism	References
BMP3 (bone morphogenetic protein 3)	Primary_assembly 32: 5,237,314 to 5,263,865 forward strand. ROS_Cfam_1.0:CM025131.1	Production of bone morphogenetic protein 3.	Increase in brain/skull size	Associated with brain and skull size.	Marchant et al. (2017)
MSRB3 (methionine sulfoxide reductase B3)	Primary_assembly 10: 7,971,175 to 8,150,414 forward strand. ROS_Cfam_1.0:CM025109.1	Catalyzes methionine sulfoxide to methionine.	Increased brain volume.	The methionine sulfoxide reductase B3 gene has been found to be associated with brain volume in a variety of studies and across a variety of species. Importantly, this gene is also associated with a variety of other neuronal functions, and ear morphology and mutations within MSRB3 are associated with human deafness, which may complicate selection directionality.	Ahmed et al. (2011); Schoenebeck et al. (2012); Hibar et al. (2017); Smith et al. (2019); Shan et al. (2021)
RUNX2 (RUNX family transcription factor 2)	Primary_assembly 12: 14,117,254 to 14,450,732 forward strand. ROS_Cfam_1.0:CM025111.1	Production of nuclear protein and transcription factors	Increased brain and skull size.	Associated with brain and skull size.	Marchant et al. (2017)

Listed from left to right are the names of the candidate gene and associated gene abbreviation, the location of the gene in the domestic dog genome as written in the Ensembl Genome Broswer, a brief synopsis of relative gene function, our hypothesized selective direction, the reasoning for why the gene may be under selection, and relevant references.

#### Problem Solving, Memory, and Learning

Urban environments may relieve selection pressures that favor complex cognition. A constant supply of anthropogenic subsidies could increase resource predictability, reducing the need to learn and problem solve to find food (Jordan et al. 2023). Thus, urbanization may impose selection pressures similar to that of domestication (Kruska 1988; Wilkins et al. 2014; Garamszegi et al. 2023). Additionally, apex predators such as mountain lions and wolves have been extirpated from developed areas, thereby relaxing the demand for antipredator problem solving strategies (Kruska 1988; Garamszegi et al. 2023). Alternatively, learning and communicating information socially about novel dangers in urban regions such as interactions with people and domestic animals or avoiding vehicles may increase the need for cognitive capacity and social learning (Dunbar 1998; Grabowski et al. 2023). For example, many bird species in urban areas quickly learn and pass on information about dangerous individual humans (Griffin and Boyce 2009; Levey et al. 2009; Cornell et al. 2012). Novel interactions with other species like people or domestic animals or novel anthropogenic infrastructure may likewise require problem-solving skills that would increase cognitive demand (Goumas et al. 2020; Lee and Thornton 2021). Environmental complexity also favors learning and cognitive ability (Dridi and Lehmann 2016). Genes that influence memory may be under selection, but directionality is similarly challenging to predict (Table 11).

**Table 11:** Candidate genes related to problem solving, memory, and learning

Candidate gene	Gene location (domestic dog)	Gene function	Selection direction	Mechanism	References
BDNF (brain-derived neurotrophic factor)	Primary_assembly 21: 49,336,157 to 49,347,969 reverse strand. ROS_Cfam_1.0:CM025120.1	BDNF encodes for a neurotrophic factor.	Altered learning and memory capabilities.	BDNF is also associated with learning and memory in domestic dogs.	Seifi Moroudi et al. (2014); Bathina and Das (2015); Santos de Sousa Fernandes et al. (2020)
CCK (cholecystokinin)	Primary_assembly 23: 11,701,991 to 11,707,673 reverse strand. ROS_Cfam_1.0:CM025122.1	Encodes for peptide hormones that act as neurotransmitters.	Changes in cognitive ability related genes.	Expressed in domestic dog hippocampus and may be associated with learning and memory.	Seifi Moroudi et al. (2014)
HACD1 (3-hydroxyacyl-CoA dehydratase 1)	Primary_assembly 2: 19,652,098 to 19,674,773 forward strand. ROS_Cfam_1.0:CM025101.1	Metabolizes fatty acids	Increased long-term memory.	HACD1 is involved in long-term memory.	Morrill et al. (2023)
HS3ST5 (heparan sulfate-glucosamine 3-sulfotransferase 5)	Primary_assembly 12: 71,020,288 to 71,268,524 reverse strand. ROS_Cfam_1.0:CM025111.1	Produces proteins that transfer sulfates.	Altered physical reasoning.	SNPs in HS3ST5 are associated with physical reasoning in domestic dogs.	Gnanadesikan et al. (2020)
OR52E2 (olfactory receptor family 52 subfamily E member 2)	Primary_assembly 21: 28,165,964 to 28,166,902 reverse strand. ROS_Cfam_1.0:CM025120.1	Encodes for an olfactory receptor.	Altered physical reasoning.	SNPs in OR52E2 are associated with physical reasoning in domestic dogs.	Gnanadesikan et al. (2020)
TAC1 (tachykinin 1)	Primary_assembly 14: 22,450,958 to 22,459,198 forward strand. ROS_Cfam_1.0:CM025113.1	Encodes for peptide hormones that act as neurotransmitters.	Changes in cognitive ability related genes.	Expressed in domestic dog hippocampus and may be associated with learning and memory.	Seifi Moroudi et al. (2014)

Listed from left to right are the names of the candidate gene and associated gene abbreviation, the location of the gene in the domestic dog genome as written in the Ensembl Genome Broswer, a brief synopsis of relative gene function, our hypothesized selective direction, the reasoning for why the gene may be under selection, and relevant references.

#### Cognitive Plasticity

Cognitive or neural plasticity refers to the brain's ability to adapt, create new tissue, and/or alter function in response to different events or stimuli (Pearson-Fuhrhop and Cramer 2010; von Bernhardi et al. 2017). This plasticity is crucial for adapting to new environments, learning, overcoming trauma, or healing in response to brain injuries (Raymont and Grafman 2006; Cauchoix et al. 2020). For instance, coyote cognitive plasticity may be selected for when dealing with brain injury as a result from vehicular collision or aggressive encounters with conspecifics, domestic animals, or humans. Additionally, for coyotes dispersing into urban areas, increased cognitive plasticity may be beneficial as individuals cope with rapidly changing environments along urbanization gradients. Thus, genes that influence the capacity for plasticity may be under positive selection in urban regions, where coyotes are likely to encounter novel items/environments and more likely to suffer brain-related injuries as a result of vehicle collisions (Bateman and Fleming 2012; Table 12).

**Table 12:** Candidate genes related to cognitive plasticity

Candidate gene	Gene location (domestic dog)	Gene function	Selection direction	Mechanism	References
GDNF (glial cell derived neurotrophic factor)	Primary_assembly 4: 71,487,859 to 71,508,270 forward strand. ROS_Cfam_1.0:CM025103.1	Production of a ligand associated with activation and recruitment of SMAD family transcription factors. Associated with survival and differentiation of neuron types.	Increased capacity for neuroplasticity.	GDNF is associated with neuroplasticity in human and animal models.	Santos de Sousa Fernandes et al. (2020)
NGF (nerve growth factor)	Primary_assembly 17: 53,781,750 to 53,782,527 reverse strand. ROS_Cfam_1.0:CM025116.1	Encodes proteins that can stimulate nerve growth.	Increased capacity for neural plasticity.	NGF is associated with neural plasticity in human and animal models.	Santos de Sousa Fernandes et al. (2020)
NGFR (nerve growth factor receptor; also known as P75NTR)	Primary_assembly 9: 26,396,704 to 26,416,363 forward strand. ROS_Cfam_1.0:CM025108.1	Encodes for the receptor that nerve growth factor binds to.	Increased capacity for neural plasticity.	P75NTR is associated with neural plasticity in human and animal models.	Santos de Sousa Fernandes et al. (2020)
TrkB (also known as EFNA5)	Primary_assembly 3: 4,169,390 to 4,439,740 forward strand. ROS_Cfam_1.0:CM025102.1	Encodes for protein associated with the prevention of axon bundling and nervous system development and differentiation.	Increased neural plasticity.	TrkB is associated with neural plasticity in human and animal models.	Santos de Sousa Fernandes et al. (2020)
BDNF (brain-derived neurotrophic factor)	Primary_assembly 21: 49,336,157 to 49,347,969 reverse strand. ROS_Cfam_1.0:CM025120.1	BDNF encodes for a neurotrophic factor.	Increased neural plasticity.	BDNF is associated with neural plasticity in human and animal models.	Seifi Moroudi et al. (2014); Bathina and Das (2015); Santos de Sousa Fernandes et al. (2020)

Listed from left to right are the names of the candidate gene and associated gene abbreviation, the location of the gene in the domestic dog genome as written in the Ensembl Genome Broswer, a brief synopsis of relative gene function, our hypothesized selective direction, the reasoning for why the gene may be under selection, and relevant references.

### Behavior

Research across taxa suggests that urban environments may favor behaviors that differ from those in nonurban environments (Ouyang et al. 2018; Caspi et al. 2022). Specifically, increased boldness, exploration tendency, and decreased aggression may confer fitness benefits for urban individuals (Sih et al. 2011; see Personality section). The process and theory of domestication (i.e. the coevoutionary process in which a species manages the survival and reproduction of another species; Purugganan 2022) may offer insights into how urban organisms adapt to life in cities: urban wildlife populations generally experience relaxed selection from natural enemies and increased selection for tolerance of humans, resulting in behaviors similar to those observed in domesticated species (Beckmann et al. 2022). In many ways, urban areas mirror the landscape of human socialization that wolves would have encountered early on in domestication (Beckmann et al. 2022; see Sociability, Range Size, and Dispersal section). It is important to note that urban animals are not being domesticated, but merely facing similar selective environments where certain behavioral traits may be favored. However, behavior is very plastic and the extent to which behavioral traits in urban populations are heritable needs further study.

#### Personality

Defined as consistent individual differences in behaviors (Sih et al. 2004; Laskowski et al. 2022), axes of personality, such as boldness, exploration, aggression, and docility, can pose ecological and evolutionary consequences in the context of urban environments (Wolf and Weissing 2012; Caspi et al. 2022). Urban coyotes can be bolder (i.e. riskier) and more willing to explore novel environments/objects than their nonurban counterparts (Breck et al. 2019; Brooks et al. 2020; Mortin et al. 2023). Although increased boldness and exploration allow individuals to exploit novel resources, these behavioral shifts can also be maladaptive if bolder and more exploratory individuals are lethally removed from populations due to conflict with people (Schell et al. 2021). Similar to boldness, aggression (i.e. agonistic reactions toward individuals) and docility (i.e. an individual's response to being handled, sometimes referred to as “tameness”) may also be under selection due to domestication-adjacent processes and/or lethal removal (Beckmann et al. 2022). This has potentially been documented in Apennine brown bears (*Ursus arctos arctos*) in Italy where no known human attacks have occurred in the past century despite cohabitation. These bears show significant enrichment for fixed differences in genes associated with tameness in other mammals (Benazzo et al. 2017). A long-term study on the process of domesticating foxes for less aggressive behavior revealed that tamer foxes showed differences in the activity of their hypothalamic–pituitary–adrenal (HPA) axis, specifically a reduction in endorphins, cortisol, adrenaline, adrenocorticotropic hormones, and proopiomelanocortin (Trut et al. 2012, 2013). They also showed different activity of neurotransmitters such as serotonin and dopamine, which can assist in regulating aggressive and docile behaviors (Trut et al. 2012, 2013). Thus, like other canids, urban coyotes may have genetic differences related to docility and aggression, especially if interbreeding with domestic dogs contributes genes linked to hypersociability (vonHoldt et al. 2017; Caragiulo et al. 2022). However, the genetic links to personality are only beginning to be understood and personality is also influenced by nongenetic early life experiences, plasticity, parental effects, and epigenetics (Sih et al. 2004; Bounduriansky and Day 2009; Schell 2018; Table 13).

**Table 13:** Candidate genes related to personality

Candidate gene	Gene location (domestic dog)	Gene function	Selection direction	Mechanism	References
BACE1 (beta-secretase 1)	Primary_assembly 5: 16,239,793 to 16,264,160 forward strand. ROS_Cfam_1.0:CM025104.1	Production of beta-secretase 1 protein. Important in the formation of myelin sheaths in peripheral nerve cells.	Altered boldness H1: Increased boldness relative to nonurban H2: Within cities, selection for intermediate-level boldness Increased or decreased aggression relative to nonurban. H1: High population densities heighten intraspecific competition, selecting for increased intraspecific aggression. H2: Food subsidies relax competition and aggression individuals are lethally removed by people, selecting for decreased aggression.	In transgenic mice, the beta-secretase 1 gene was found to be significantly correlated with boldness, anxiousness, and increased 5-hydroxytryptamine turnover.	Harrison et al. (2003); Hu et al. (2006)
CD36 (cluster of differentiation 36)	Primary_assembly 18: 20,542,818 to 20,821,688 forward strand. ROS_Cfam_1.0:CM025117.1	Production of glycoprotein on platelets certain cell types.	See BACE1 predictions	CD36 on chromosome 18 is associated with canine aggression and fear toward new conspecifics and humans and is highly selected for in dogs.	Zapata et al. (2016)
DRD1/D4 (dopamine receptors D1 and D4)	Primary_assembly 4: 37,916,400 to 37,921,094 forward strand. ROS_Cfam_1.0:CM025103.1 Primary_assembly 18: 26,314,085 to 26,316,803 forward strand. ROS_Cfam_1.0:CM025117.1	Production of dopamine receptor protein.	See BACE1 predictions	Previous work on domestic dogs highlighted the dopamine receptors D1 and D4 as important for canine aggression.	Våge et al. (2010); Proskura et al. (2013)
GNAT3 (G protein subunit alpha transducin 3)	Primary_assembly 18: 20,619,251 to 20,675,853 reverse strand. ROS_Cfam_1.0:CM025117.1	…	See BACE1 predictions	GNAT3 on chromosome 18 is associated with canine aggression and fear toward new conspecifics and humans and is highly selected for in dogs.	Zapata et al. (2016)
GRM8 (glutamate metabotropic receptor 8)	Primary_assembly 14: 8,755,412 to 9,493,659 forward strand. ROS_Cfam_1.0:CM025113.1	Production of an excitatory neurotransmitter.	See BACE1 predictions	The glutamate metabotropic receptor 8 gene is associated with stranger-directed aggression and has been indicated as one of the more important genes for canine domestication. This gene has undergone significant selection during the domestication of wolves and in humans.	Wang et al. (2013); Hare (2017); MacLean et al. (2019)
HMGA2 (high mobility group AT-hook 2)	Primary_assembly 10: 8,459,180 to 8,601,880 forward strand. ROS_Cfam_1.0:CM025109.1	Production of a protein important in cellular response to stimuli	Decreased aggression toward people.	HMGA2 is highly selected for in dogs and is associated with aggression and fear toward new conspecifics and humans.	Zapata et al. (2016)
HTR1D (5-hydroxytryptamine receptor 1D)	Primary_assembly 2: 76,740,524 to 76,761,502 forward strand. ROS_Cfam_1.0:CM025101.1	Many functions including affecting serotonin and anxiety	See BACE1 prediction.	In domestic dogs, the 5-hydroxytryptamine receptor 1D gene is associated with bold behavior. Previous work on domestic dogs highlighted the HTR1D as important for canine aggression as well.	Våge et al. (2010); Hall and Wynne (2012); Proskura et al. (2013)
HTR2C (5-hydroxytryptamine receptor 2C)	Primary_assembly X: 89,055,355 to 89,357,011 forward strand. ROS_Cfam_1.0:CM025138.1	Many functions including affecting serotonin and anxiety	See BACE1 predictions	Previous work on domestic dogs highlighted the 5-hydroxytryptamine receptor 2C gene as important for canine aggression.	Våge et al. (2010); Proskura et al. (2013)
IGF1 (insulin-like growth factor 1)	Primary_assembly 15: 41,856,552 to 41,930,595 reverse strand. ROS_Cfam_1.0:CM025114.1	Production of the hormone insulin-like growth factor 1. Involved in body size determination across mammalian species.	Aggression: See BACE1 predictions	IGF1 is highly selected for in dogs and is associated with aggression and fear toward new conspecifics and humans.	Favier et al. (2001); Sutter et al. (2007); Zapata et al. (2016); Plassais et al. (2019)
LRRTM4 (leucine-rich repeat transmembrane neuronal 4)	Primary_assembly 17: 46,912,071 to 47,618,737 forward strand. ROS_Cfam_1.0:CM025116.1	Regulates synapse assembly among other functions.	Decrease in the need to prey on animals due to anthropogenic food supplementation as well as the potential for increased conflict surrounding predation of domestic animals may select for individuals with less prey drive.	LRRTM4 is associated with predation behavior in domestic dogs.	Shan et al. (2021)
PCDH9 (protocadherin 9)	Primary_assembly 22: 21,579,414 to 22,465,684 reverse strand. ROS_Cfam_1.0:CM025121.1	Production of protocadherin, a transmembrane protein.	See HTR1D predictions	In domestic dogs, the protocadherin 9 gene is associated with bold behavior. SNPs within this gene may be of particular interest to those studying boldness in urban coyotes.	Hall and Wynne (2012)
PDE7B (phosphodiesterase 7B)	Primary_assembly 1: 28,635,503 to 28,950,201 forward strand. ROS_Cfam_1.0:CM025100.1	Many functions including modulating dopamine pathways and metabolism of cyclic adenosine monophosphate and cyclic guanosine monophosphate.	See BACE1 predictions	In foxes, PDE7B is associated with tameness and aggression and underwent strong selection during dog domestication	Hekman et al. (2018); MacLean et al. (2019)
SLC1A2, SLC6A1 (solute carrier family 1 member 2, family 6 member 1)	Primary_assembly 20: 7,371,116 to 7,413,202 reverse strand. ROS_Cfam_1.0:CM025119.1	Encodes for solute transporter proteins	See HTR1D predictions	Previous work on domestic dogs highlighted the solute carrier family 6 member 1 gene, as important for canine aggression.	Våge et al. (2010); Proskura et al. (2013)

Listed from left to right are the names of the candidate gene and associated gene abbreviation, the location of the gene in the domestic dog genome as written in the Ensembl Genome Broswer, a brief synopsis of relative gene function, our hypothesized selective direction, the reasoning for why the gene may be under selection, and relevant references.

#### Sociability, Range Size, and Dispersal

High habitat fragmentation in cities coupled with increased food availability can reduce coyote home range sizes and increase population densities (Gehrt 2007). The decreased physical space between nonrelated coyotes may necessitate an increased tolerance of competitors. However, little is known about how urbanization affects the behavior of organisms toward conspecifics and other species, including whether changes in intra- and interspecies tolerance are adaptive, neutral, or maladaptive (Łopucki et al. 2021).

Urbanization has also been documented to alter coyote dispersal timing and distance (Zepeda et al. 2021). Many organisms show a discernible genetic foundation for dispersal behavior (Saastamoinen et al. 2018), and the concept of “spatial personalities,” consistent individual differences in spatial behavior, as being genetically or culturally inherited has recently emerged (Spiegel et al. 2017; Stuber et al. 2022). Genes associated with spatial personalities or range expansion may thus be worthwhile to review when considering dispersal patterns (Heppenheimer et al. 2018). Importantly, the physical layout of each individual city and the distribution of available habitat within a city may have particular impacts on range size and dispersal distances (Table 14).

**Table 14:** Candidate genes related to sociability, range size, and dispersal

Candidate gene	Gene location (domestic dog)	Gene function	Selection direction	Mechanism	References
ALK (anaplastic lymphoma kinase)	Primary_assembly 17: 23,572,834 to 24,250,519 reverse strand. ROS_Cfam_1.0:CM025116.1	Production of a tyrosine kinase.	Altered dispersal and range size.	Associated with range expansion in coyotes.	Heppenheimer et al. (2018)
CACNA1C (calcium voltage-gated channel subunmit alpha1 C)	Primary_assembly 27: 44,471,385 to 45,116,929 forward strand. ROS_Cfam_1.0:CM025126.1	Production of calcium channel	Altered dispersal and range size.	Associated with range expansion in coyotes.	Heppenheimer et al. (2018)
EML1 (echinoderm microtuble-associated protein-like 1)	Primary_assembly 8: 68,391,815 to 68,580,640 forward strand. ROS_Cfam_1.0:CM025107.1	Enables microtubule binding activity and is involved in brain development.	Altered inhibitory control	EML1 is associated with inhibitory control in domestic dogs.	Gnanadesikan et al. (2020)
EPHA6 (ephrin type-A receptor 6)	Primary_assembly 33: 4,624,891 to 4,893,790 forward strand. ROS_Cfam_1.0:CM025132.1	Production of ephrin type A receptor 6 implicated in between cell communication.	Altered dispersal and range size.	Associated with range expansion in coyotes.	Heppenheimer et al. (2018)
GTF2I (general transcription factor IIi) GTF2IRD1 (general trasncription facotr IIi domain-containing protein 1)	Primary_assembly 6: 5,543,753 to 5,656,989 reverse strand. ROS_Cfam_1.0:CM025105.1 Primary_assembly 6: 5,704,056 to 5,814,824 reverse strand. ROS_Cfam_1.0:CM025105.1	Production of a phosphoprotein.	Altered sociability with humans.	Variants in GTF2I and GTF2IRD1 are associated with hypersociability in humans and domestic dogs.	vonHoldt et al. (2017)
HS6ST2 (heparan sulfate 6-O-sulfotransferase 2)	Primary_assembly X: 105,389,174 to 105,672,467 reverse strand. ROS_Cfam_1.0:CM025138.1	Catalyzes conversion of sulfate to heparan sulfate.	See HACD1 predictions.	In domestic dogs, HS6ST2 is associated with sociability.	Zapata et al. (2016)
HACD1 (3-hydroxyacyl-CoA dehydratase 1)	Primary_assembly 2: 19,652,098 to 19,674,773 forward strand. ROS_Cfam_1.0:CM025101.1	Metabolizes fatty acids	Altered sociability with humans. Increased tolerance of people.	In domestic dogs, a region downstream of HACD1 is associated with increased sociability with human beings.	Morrill et al. (2023)

Listed from left to right are the names of the candidate gene and associated gene abbreviation, the location of the gene in the domestic dog genome as written in the Ensembl Genome Broswer, a brief synopsis of relative gene function, our hypothesized selective direction, the reasoning for why the gene may be under selection, and relevant references.

#### Nocturnality

In urban areas, wildlife tends to shift their activity nocturnally in response to diurnal human activity (Gaynor et al. 2018) and artificial light at night (Beier 2006). Changes in diel activity can adversely affect energy metabolism (Jha et al. 2015), but the metabolic consequences of shifts in diel activity are poorly characterized (see Metabolic Rate and Function sections in Diet section). These shifts toward nocturnality could lead to morphological changes, particularly in eye and skull shape, in ways that enhance fitness in nighttime conditions (Hall et al. 2012). On the other hand, despite increased nocturnality, the additional light from artificial light sources prevalent in urban areas may reduce the advantage of any morphological features that enhance low-light vision (Table 15).

**Table 15:** Candidate genes related to nocturnality

Candidate gene	Gene location (domestic dog)	Gene function	Selection direction	Mechanism	References
CLOCK (circadian locomotor output cycles kaput)	Primary_assembly 13: 48,297,870 to 48,436,440 reverse strand. ROS_Cfam_1.0:CM025112.1	Gene that produces proteins with DNA histone acetyltransferase activity	Altered circadian rhythm via increased nocturnality.	To avoid human activity, urban wildlife often becomes more nocturnal. This shift in behavior may select for changes in circadian rhythm related genes.	Wager-Smith and Kay (2000)
CNGA1, CNGA3 (cyclic nucleotide-gated channel subunit alpha 1 and 3)	Primary_assembly 13: 44,430,868 to 44,464,133 reverse strand. ROS_Cfam_1.0:CM025112.1 Primary_assembly 10: 45,104,784 to 45,151,081 reverse strand. ROS_Cfam_1.0:CM025109.1	Production of ion channels that are for cellular response to stimuli.	Increased visual acuity.	CNGA1 and 3 are involved in low-light vision.	Wu et al. (2017)
CRY1/CRY2 (cytochrome circadian regulator 1/2)	Primary_assembly 10: 32,522,596 to 32,623,626 forward strand. ROS_Cfam_1.0:CM025109.1 Primary_assembly 18: 44,194,116 to 44,229,483 reverse strand. ROS_Cfam_1.0:CM025117.1	Produces proteins that are key components of the circadian core oscillator complex.	Altered circadian rhythm via increased nocturnality.	See CLOCK mechanism.	Wager-Smith and Kay (2000)
DBT (dihydrolipoadmide branched chain transacylase)	Primary_assembly 6: 50,101,072 to 50,153,426 forward strand. ROS_Cfam_1.0:CM025105.1	Production of an enzyme associated with breakdown of branched-chain amino acids.	Altered circadian rhythm via increased nocturnality.	See CLOCK mechanism.	Wager-Smith and Kay (2000)
GRK1 (G protein-coupled receptor kinase 1)	Primary_assembly 22: 61,596,581 to 61,609,597 forward strand. ROS_Cfam_1.0:CM025121.1	Production of a protein responsible for rhodopsin deactivation.	Increased visual acuity.	GRK1 is involved in low-light vision.	Wu et al. (2017)
GUCY2D (guanylate cyclase 2D)	Primary_assembly 5: 32,946,524 to 32,962,025 forward strand. ROS_Cfam_1.0:CM025104.1	Production of guanylate cyclase protein that is essential for vision.	Increased visual acuity.	GUCY2D is involved in photoresponse recovery. These gene went under heavy positive selection when ancestral mammals entered dim-light environments and may have implications for nocturnality.	Wu et al. (2017)
PDE6A/B (phosphodiesterase 3A and 3B)	Primary_assembly 4: 59,584,982 to 59,644,927 forward strand. ROS_Cfam_1.0:CM025103.1 Primary_assembly 3: 92,746,065 to 92,774,246 reverse strand. ROS_Cfam_1.0:CM025102.1	Production of phosphodiesterase expressed in rod cells.	Increased visual acuity.	PDE6A/B are involved in low-light vision.	Wu et al. (2017)
PER2 (period circadian clock 2)	Primary_assembly 25: 49,102,151 to 49,143,375 reverse strand. ROS_Cfam_1.0:CM025124.1	A period family gene that is expressed in circadian patterns.	Altered circadian rhythm via increased nocturnality.	See CLOCK mechanism.	Wager-Smith and Kay (2000)
RGS9 (regulator of G protein signaling 9)	Primary_assembly 9: 16,472,820 to 16,568,043 reverse strand. ROS_Cfam_1.0:CM025108.1	Production of a signaling protein important pathway activation.	Increased visual acuity.	RGS9 is involved in photoresponse recovery and inactivation of transducin.	Wu et al. (2017)
RH1 (rhodopsin 1)	NA	Production of rod-specific rhodopsin.	Increased visibility in low light.	RH1 is involved in photon absorption.	Wu et al. (2017)

Listed from left to right are the names of the candidate gene and associated gene abbreviation, the location of the gene in the domestic dog genome as written in the Ensembl Genome Broswer, a brief synopsis of relative gene function, our hypothesized selective direction, the reasoning for why the gene may be under selection, and relevant references.

### Reproduction and Sexual Selection

With a surplus of anthropogenic resources, urban wildlife populations generally produce larger litters compared to nonurban populations (Santini et al. 2018; see Litter Size, Estrus Timing, and Mate Selection section). The mechanisms behind this plasticity in litter size may be a reflection of reduced physiological stress associated with increased food availability (Bronson 1989; Boutin 1990; Ruffino et al 2014). Alternatively, resource availability may trigger epigenetic changes allowing for increased placental and fetal development (Nordin et al. 2014). While pup survival rate is often higher in urban regions, adult mortality is likely similar to, or higher than, that of nonurban coyotes (Riley et al. 2003; Gehrt et al. 2011; Bateman and Fleming 2012).

The additional food resources provided by urbanization may allow for more energy to go directly toward reproduction. In addition to having larger litter sizes, increased caloric intake may allow for changes in reproductive timing or allow females to come into estrus more frequently (Gittleman and Thompson 1988; see Litter Size, Estrus Timing, and Mate Selection section). Thus, we may expect that in urban environments where resources are more prevalent than in nonurban regions, genes that allow for greater plasticity in litter size may be under selection (Lambert et al. 2021). Additionally, while there is little research on mate selection in urban mammals, differences in selection pressure and exposure to different urban contaminants that can alter behavior and cognition may lead to changes in the ways coyotes select their mates (see Litter Size, Estrus Timing, and Mate Selection section). Higher densities of coyotes may also lead to differences in sociability, which could also alter mate selection. Finally, elements of the urban environment such as environmental estrogens or androgens (see Environmental Estrogens and Androgens section) and excessive heat (see Heat Stress section) may have adverse effects on reproduction and select for genes that mitigate the effects of exposure.

#### Litter Size, Estrus Timing, and Mate Selection

Wolves only come into estrus for 1 to 2 weeks/year and have a single yearly litter during the spring (Packard 2003; McNay et al. 2006). Many species, including wolves, time their reproduction so that young are born in the season with the most resource availability (Tveraa et al. 2013). In contrast, domestic dogs go into estrus on average every 7 months, can have multiple litters per year, and can have pups at any time of the year (Macdonald and Carr 1995; Boitani et al. 2006; Lord et al. 2013). With high resource availability in urban regions, there should be a less stringent need for young to be born during a specific time of the year, which may release evolutionary constraints on the timing of reproduction (Post et al. 2001). Post et al. (2001) noted that reproductive asynchrony increased with environmental disturbance, which is high in urban areas. It should be noted that much of the increased litter size seen as a result of increased food is a plastic response, but that this plasticity could be underlaid by genetic architecture (Casto-Robollo et al. 2020). While there is little research about mate selection in urban mammals, there is evidence for altered sexual selection in other urban vertebrates, especially in birds (Cronin et al. 2022). For mice and some other mammalian species, mate selection can be correlated with the major histocompatibility complex, where mates often have strong differences in the major histocompatibility complex that would confer enhanced pathogen resistance to offspring (Wedekind et al. 1995; Ober et al. 1997; Penn and Potts 1998; Yamazaki and Beauchamp 2007; Santos et al. 2016). With higher densities of coyotes in urban areas and potentially altered sociability, genes that are implicated in mate choice may be under selection or released from evolutionary constraints (Table 16).

**Table 16:** Candidate genes related to litter size, estrus timing, and mate selection

Candidate gene	Gene location (domestic dog)	Gene function	Selection direction	Mechanism	References
AGBL1 (AGBL carboxypeptidase 1)	Primary_assembly 3: 50,060,828 to 50,751,728 forward strand. ROS_Cfam_1.0:CM025102.1	Production of protein involved in glutamylation of polyglutamylated proteins.	Released constraints on reproductive timing.	In sheep, AGBL1 is associated with reproductive seasonality.	Posbergh et al. (2019)
CD151 (cluster of differentiation 151)	Primary_assembly 18: 45,860,164 to 45,864,953 forward strand. ROS_Cfam_1.0:CM025117.1	Encodes for a transmembrane protein and is involved in cellular processes such as cell motility, adhesion, and invasion.	Released constraints on reproductive timing.	CD151 is associated with reproduction in nonseasonally breeding mammalian species.	Meng et al. (2015)
CGA (glycoprotein hormones, alpha polypeptide)	Primary_assembly 12: 47,386,743 to 47,405,483 reverse strand. ROS_Cfam_1.0:CM025111.1	Production of a main glycoprotein hormone associated with reproduction.	Released constraints on reproductive timing.	CGA is associated with reproduction in nonseasonally breeding mammalian species.	Meng et al. (2015)
CMTM6 (CKLF-like MARVEL transmembrane domain containing 6)	Primary_assembly 23: 12,586,949 to 12,608,824 forward strand. ROS_Cfam_1.0:CM025122.1	A transmembrane protein, but the exact function of this gene is unknown.	Released constraints on reproductive timing.	CMTM6 is associated with reproduction in seasonally breeding mammalian species.	Meng et al. (2015)
DNAH1 (dynein axonemal heavy chain 1)	Primary_assembly 20: 37,714,969 to 37,787,882 reverse strand. ROS_Cfam_1.0:CM025119.1	Encodes for protein that provides structural support in sperm.	Released constraints on reproductive timing.	DNAH1 is associated with reproduction in nonseasonally breeding mammalian species.	Meng et al. (2015)
GDF9 (growth differentiation factor 9)	Primary_assembly 11: 21,931,265 to 21,936,196 reverse strand. ROS_Cfam_1.0:CM025110.1	Production of a ligand associated with activation and recruitment of SMAD family transcription factors and is related to ovarian function.	Released constraints on litter size.	GDF9 is associated with litter size in some domestic dog breeds.	Torrecilha et al. (2019)
HECW1 (HECT, C2 and WW domain containing E3 ubiquitin protein ligase 1)	Primary_assembly 18: 6,690,496 to 7,130,777 reverse strand. ROS_Cfam_1.0:CM025117.1	Production of protein associated with ubiquitin protein ligase among other functions.	Released constraints on litter size.	In sheep HECW1 is associated with litter size.	Tao et al. (2021)
HTR1E (5-hydroxytryptamine receptor 1E)	Primary_assembly 12: 47,244,335 to 47,327,888 forward strand. ROS_Cfam_1.0:CM025111.1	Enables serotonin receptor and binding activity.	Released constraints on litter size.	In sheep HTR1E is associated with litter size.	Tao et al. (2021)
INVS (inversin)	Primary_assembly 11: 58,161,184 to 58,364,961 forward strand. ROS_Cfam_1.0:CM025110.1	Produces a protein with ankyrin domains.	Released constraints on reproductive timing.	INVS is associated with reproduction in nonseasonally breeding mammalian species.	Meng et al. (2015)
MSANTD1 (Myb/SANT DNA binding domain containing 1; also known as HTT)	Primary_assembly 3: 61,536,790 to 61,550,600 reverse strand. ROS_Cfam_1.0:CM025102.1	Involved in transcription regulation.	Increased litter size.	MSANTD1 is associated with litter size in domestic dogs.	Smith et al. (2019)
MSRB3 (methionine sulfoxide reductase B3)	Primary_assembly 10: 7,971,175 to 8,150,414 forward strand. ROS_Cfam_1.0:CM025109.1	Catalyzes methionine sulfoxide to methionine.	Increased litter size.	MSRB3 is associated with litter size in domestic dogs.	Ahmed et al. (2011); Schoenebeck et al. (2012); Hibar et al. (2017); Smith et al. (2019); Shan et al. (2021)
NELFCD (negative elongation factor complex member C/D; also known as TH1L)	Primary_assembly 24: 44,596,635 to 44,609,369 forward strand. ROS_Cfam_1.0:CM025123.1	Produces proteins that repress transcriptional elongation by RNA polymerase II.	Released constraints on reproductive timing.	NELFCD (also known as TH1L) is associated with reproduction in seasonally breeding mammalian species.	Meng et al. (2015)
THRAP3 (thyroid hormone receptor-associated protein 3)	Primary_assembly 15: 6,090,819 to 6,157,976 reverse strand. ROS_Cfam_1.0:CM025114.1	Encodes for a hormone binding receptor that is associated with metabolism and circadian rhythm.	Released constraints on reproductive timing.	THRAP3 is associated with reproduction in seasonally breeding mammalian species.	Meng et al. (2015)

Listed from left to right are the names of the candidate gene and associated gene abbreviation, the location of the gene in the domestic dog genome as written in the Ensembl Genome Broswer, a brief synopsis of relative gene function, our hypothesized selective direction, the reasoning for why the gene may be under selection, and relevant references.

#### Heat Stress

Reproduction is particularly susceptible to heat stress and can be affected at any stage from gamete production to raising young after birth (Fuller et al. 2020). For coyotes, external testes make male gamete production particularly prone to negative outcomes from excessive heat exposure (Boni 2019). Increased heat stress can lead to a reduction in sperm count, genetic material within gametes, and production of various sex hormones (Rahman et al. 2018; Fuller et al. 2020). While coyotes can reduce heat stress through behavioral means like moving to shady areas, even small increases in temperature to testes can affect sperm production. For instance in humans, even sitting for a few extra hours or wearing tight pants can significantly reduce sperm count and quality, demonstrating the extreme heat sensitivity of external mammalian testes (Durairajanayagam et al. 2014). Thousands of genes go into the production and maintenance of healthy and functioning sperm, and the expression of these genes has been shown to change within the testes of different species before and after heat stress (Song et al. 2022). While these changes to expression are likely epigenetic rather than genetic changes, there are select genes that may be of particular interest when examining heat resistance in spermatogenesis (Table 17).

**Table 17:** Candidate genes related to heat stress

Candidate gene	Gene location (domestic dog)	Gene function	Selection direction	Mechanism	References
BRCA1 (breast cancer gene 1)	Primary_assembly 9: 20,677,128 to 20,743,989 forward strand. ROS_Cfam_1.0:CM025108.1	Many functions: DNA repair, transcriptional activation, cell cycle regulation and chromatin remodeling	Increased spermatogenesis thermotolerance.	This gene is involved in spermatogenesis. Variants that affect sperm heat tolerance and viability may be advantageous in high heat stress environments such as in urban heat islands.	Gorodetska et al. (2019); Song et al. (2022)
BRDT (bromodomain testis associated)	Primary_assembly 6: 57,301,727 to 57,347,925 reverse strand. ROS_Cfam_1.0:CM025105.1	Production of bromodomain testis-specific protein	Increased spermatogenesis thermotolerance.	This gene is involved in spermatogenesis. Variants that affect sperm heat tolerance and viability may be advantageous in high heat stress environments such as in urban heat islands.	Song et al. (2022)
CEP120 (centrosomal protein 120)	Primary_assembly 11: 13,894,248 to 14,011,305 reverse strand. ROS_Cfam_1.0:CM025110.1	Production of centrosomal protein 120. Important in maintaining centrosome homeostasis.	Increased spermatogenesis thermotolerance.	This gene is involved in spermatogenesis. Variants that affect sperm heat tolerance and viability may be advantageous in high heat stress environments such as in urban heat islands.	Mahjoub et al. (2010); Song et al. (2022)
SYCP2 (synaptonemal complex protein 2)	Primary_assembly 24: 45,337,858 to 45,398,458 reverse strand. ROS_Cfam_1.0:CM025123.1	Produces synaptonemal complex protein 2 implicated in attaching homologous chromosomes during meiosis.	Increased spermatogenesis thermotolerance.	This gene is involved in spermatogenesis. Variants that affect sperm heat tolerance and viability may be advantageous in high heat stress environments such as in urban heat islands.	Song et al. (2022)
TDRD9 (tudor domain containing 9)	Primary_assembly 8: 71,888,154 to 71,992,748 forward strand. ROS_Cfam_1.0:CM025107.1	Production of tudor domain implicated in spermatogenesis and RNA binding activity.	Increased spermatogenesis thermotolerance.	This gene is involved in spermatogenesis. Variants that affect sperm heat tolerance and viability may be advantageous in high heat stress environments such as in urban heat islands.	Song et al. (2022)

Listed from left to right are the names of the candidate gene and associated gene abbreviation, the location of the gene in the domestic dog genome as written in the Ensembl Genome Broswer, a brief synopsis of relative gene function, our hypothesized selective direction, the reasoning for why the gene may be under selection, and relevant references.

#### Environmental Estrogens and Androgens

Environmental estrogens and androgens, a category of endocrine disruptors, are of great concern in urban centers (Croteau et al. 2008; Schultz et al. 2013). Environmental estrogens and androgens can affect sex determination, pubescence, carcinogenesis, cognition, and fertility (Sonnenschein and Soto 1998; Gonsioroski et al. 2020). While more frequently studied as a source of harm for amphibians and fishes, these endocrine disruptors can also have negative effects on mammalian species (Croteau et al. 2008; Gonsioroski et al. 2020; Pocar et al. 2023). In mice, studies have found that certain mouse strains have reduced effects from particular estrogenic compounds, suggesting a potential genetic or epigenetic source of resistance. For instance, mouse strains that have been selected for large litter size showed little to no response to large doses of 17β-estradiol, while other lines showed inhibition of spermatid maturation. Other studies have found specific genes correlated to environmental estrogen resistance (Spearow et al. 1999; Stenz et al. 2019). If coyotes are frequently exposed to these environmental estrogens and androgens and they begin to have substantial negative fitness or reproductive effects, selection for genes or epigenetic regulation that improves resistance to, or reduces effects of, these compounds may occur (Table 18).

**Table 18:** Candidate genes related to environmental estrogens and androgens

Candidate gene	Gene location (domestic dog)	Gene function	Selection direction	Mechanism	References
ESR1 (estrogen receptor 1)	Primary_assembly 1: 42,267,396 to 42,570,184 forward strand. ROS_Cfam_1.0:CM025100.1	Production of estrogen receptor and ligand-activated transcription factor	Increased environmental estrogen resistance.	Mice resistant to DEHP (an environmental estrogen) were associated with a modified ligand binding site due to a SNP in the estrogen receptor 1 gene and increased expression of this SNP-affected ESR1 variant 4.	Weise et al. (2001); Plassais et al. (2019); Stenz et al. (2019)
FOXA3 (forkhead box A3)	Primary_assembly 1: 110,281,781 to 110,290,035 reverse strand. ROS_Cfam_1.0:CM025100.1	Produces hepatocyte nuclear factors that act as transcription activators.	Increased environmental estrogen resistance.	Mice that were resistant to DEHP (an environmental estrogen) were associated with the absence of forkhead box A3 RNA.	Chen et al. (2014); Stenz et al. (2019)
SVS2 through 6 (seminal vesicle secretory protein)	Primary_assembly 17: 60,182,738 to 60,196,859 reverse strand. ROS_Cfam_1.0:CM025116.1	Encodes for seminal vesicle proteins.	Increased environmental estrogen resistance.	Studies of similar estrogenic compounds in mice have found five SNPs in sex steroid hormone signaling pathways associated with susceptibility to a specific estrogenic compound (DEHP) and silencing of six seminal vesicle protein genes (Svs2, Svs3a, Svs3b, Svs4, Svs5, and Svs6).	Stenz et al. (2019)

Listed from left to right are the names of the candidate gene and associated gene abbreviation, the location of the gene in the domestic dog genome as written in the Ensembl Genome Broswer, a brief synopsis of relative gene function, our hypothesized selective direction, the reasoning for why the gene may be under selection, and relevant references.

## Closing Remarks

Historically evolution was thought to occur on vast chronological scales. We now understand that evolution can happen within just a few generations, especially when selection strength is high or variable at the microhabitat scale and where behavioral or spatial isolation is present (Richardson et al. 2014; Caspi et al. 2022). Urban areas offer a unique glimpse into how evolution functions on smaller timescales and how species adapt to human presence and novel environments. Importantly, prior to making any conclusions on selection, future studies must make an effort to understand genetic drift and gene flow in their study area. Gene flow among urban and nonurban coyotes may swamp any potentially advantageous alleles for urban living, thereby inhibiting selection for urban-specific adaptations. Similarly, it is important to understand the potential for current or past hybridization events between coyotes, wolves, and domestic dogs, which may muddy selection pressures and introduce novel characteristics not inherent to coyotes (Caragiulo et al. 2022).

In this manuscript, we outlined several pathways by which urban pressures may shape the evolution of urban coyotes, a common North American urban mammal. We specifically focused on five categories that are the most evolutionarily relevant to our focal species: (i) diet; (ii) immunology, detoxification, and thermoregulation; (iii) personality; (iv) cognition and neuroanatomy; and (v) reproduction and sexual selection. We hope that this may serve as a guideline for scientists researching urban adaptation in coyotes, as well as a starting point for urban evolutionary biologists studying other species to create their own candidate gene catalog.

While we have seen continued growth in the field of urban evolution (Rivkin et al. 2018), linking specific genes to adaptation in urban regions is still relatively unexplored. Linking phenotype to genotype is a primary focus in evolutionary biology, and recent advances in our understanding of gene conservation, as well as DNA sequencing technology, make the candidate gene approach especially relevant for current urban evolution research. Additionally, because many species under selection in cities are nonmodel organisms, this approach can be leveraged to determine if and how genes influence urban phenotypes. While some work has shown direct links between a genotype and an adaptive urban phenotype (Whitehead et al. 2017), more work is needed to determine if the same genes are under selection across organisms and among cities or if adaptations are the result of epigenetics or plasticity. We anticipate the next decade will provide numerous novel urban adaptation studies on nonmodel systems in cities, with more of these studies directly linking phenotype and genotype.

## Data Availability

No data were collected for this study, and all resources and references are listed within the text.
